# Techniques for mitigating overfitting in machine learning: a comprehensive review, taxonomy, and practical guide

**DOI:** 10.3389/frai.2026.1794271

**Published:** 2026-03-24

**Authors:** Alexander Pearson Sheppert

**Affiliations:** 1 Capitol Technology University, Laurel, MD, United States; 2 Legacy Health, Vancouver, WA, United States

**Keywords:** data augmentation, deep learning, ensembling, generalization, model selection, narrative review, overfitting, regularization

## Abstract

**Introduction:**

Overfitting remains a persistent barrier to reliable machine learning, especially in modern overparameterized deep models.

**Methods:**

We conducted a narrative review synthesizing approximately 95 core studies (1943–2026) identified through structured searches of IEEE Xplore, ACM Digital Library, arXiv, Google Scholar, and Semantic Scholar, extracting mechanisms, assumptions, and empirical evidence for prominent methods for mitigating overfitting.

**Results:**

We organize techniques into a unified five-family taxonomy (parameter-, training-, data-, ensemble-, and objective-based) and provide a practical decision framework that maps data regimes, model families, and real-world scenarios to actionable regularization strategies.

**Conclusion:**

Overfitting mitigation benefits from coordinated choices in data, model capacity, optimization, and evaluation. Our taxonomy and decision framework help practitioners select complementary interventions and avoid common pitfalls such as leakage and over-regularization.

## 1 Introduction

Machine learning has transformed many domains, from medical diagnosis and autonomous vehicles to financial forecasting and natural language understanding (Russell and Norvig, 2010; LeCun et al., 2015). The remarkable success of modern machine learning, particularly deep neural networks, stems from their capacity to learn complex, hierarchical representations from data. However, this same capacity also makes models vulnerable to overfitting, the tendency to memorize training data rather than learn patterns that generalize (Geman et al., 1992; Vapnik, 1998).

Overfitting occurs when a model captures not only the underlying signal in training data but also its idiosyncratic noise. The consequences are severe: a model exhibiting exceptional training performance may fail catastrophically on new data, rendering it unreliable for deployment. In practice, overfitting often manifests as reliance on spurious correlations. As Vigen (2015)'s catalog of spurious correlations demonstrates, statistical associations are ubiquitous; for example, the number of films Nicolas Cage appeared in correlates almost perfectly with swimming pool drownings. A model that learns such associations provides no predictive value.

The battle against overfitting has shaped machine learning research for over six decades. From Tikhonov's regularization in the 1940s (Tikhonov, 1943) to modern techniques like sharpness-aware minimization (Foret et al., 2021) and analyses of double descent (Belkin et al., 2019; Nakkiran et al., 2021), the field has developed an extensive arsenal of strategies for mitigating overfitting. This proliferation creates the practical challenge of a bewildering array of options with limited guidance on selection and combination, and with success rates that vary widely across tasks, data regimes, and evaluation protocols.

### 1.1 Motivation and scope

This survey addresses three critical gaps in the existing literature:

1. Fragmented Knowledge: Techniques for mitigating overfitting are often studied in isolation within specific subfields. Regularization methods developed for linear models, dropout-style interventions for neural networks, and ensemble methods for tree-based models are typically analyzed within distinct modeling traditions, assumptions, and evaluation norms, making it difficult to compare results or combine methods principledly.
2. Rapid Evolution: The past decade has witnessed major shifts in our understanding of generalization in modern deep learning, including double descent (Belkin et al., 2019; Nakkiran et al., 2021; Kubo et al., 2026), neural tangent kernel theory (Jacot et al., 2018), grokking (Power et al., 2022; He et al., 2026), and sharpness-aware training with recent refinements (Foret et al., 2021; Li et al., 2024). Many existing surveys predate these developments or treat them in isolation.
3. Practical Guidance Gap: While theoretical analyses abound, practitioners lack systematic guidance for selecting and combining techniques based on problem characteristics, computational constraints, and desired properties.

### 1.2 Contributions

This survey makes the following contributions:

1. Comprehensive Taxonomy: We propose a five-family taxonomy (Parameter-based, Training-based, Data-based, Ensemble-based, Objective-based) that unifies techniques across statistical learning, neural networks, and ensemble methods.
2. Comprehensive Coverage: We synthesize approximately 95 core studies spanning 1943–2026, covering foundational statistical methods through cutting-edge deep learning techniques.
3. Unified Mathematical Framework: We present a mathematical framework connecting regularization approaches through the lens of constrained optimization and Bayesian inference.
4. Modern Phenomena Integration: We incorporate recent paradigm-shifting discoveries including double descent, grokking, sharpness-aware minimization, and neural scaling laws.
5. Practical Decision Framework: We provide empirically-grounded guidelines for technique selection based on problem type, data availability, and computational resources.
6. Objective-based Analysis: We analyze the emerging paradigm of objective-based generalization, from historical risk-adjusted ratios in finance to modern objective reformulations in machine learning.

### 1.3 Paper organization

The remainder of this paper is organized as follows. Section 2 reviews related surveys. Section 3 presents our review methodology. Section 4 establishes theoretical foundations including the bias-variance decomposition, statistical learning theory, and modern perspectives. Section 5 details our five-family taxonomy with analysis of each technique. Section 6 provides comparative analysis and practical guidelines. Section 7 discusses emerging trends and future directions. Section 8 concludes the survey.

## 2 Related work

Several surveys have addressed aspects of overfitting mitigation techniques, though none provide the comprehensive, unified treatment we present here.

CNN-Focused Surveys: Santos et al. (2022) provide an extensive survey of regularization methods specifically for convolutional neural networks, categorizing techniques into data augmentation, network architecture modifications, and explicit regularization. While comprehensive for CNNs, their scope excludes classical statistical methods, ensemble techniques, and the emerging objective-based paradigm we analyze.

Deep Learning Regularization: Moradi et al. (2020) survey regularization strategies for deep models, covering dropout variants, batch normalization, and weight decay. However, their treatment predates the double descent and grokking discoveries that have fundamentally reshaped our understanding of generalization in overparameterized models.

Classical Statistical Treatments: Hastie et al. (2009)'s seminal textbook provides rigorous coverage of L1/L2 regularization, cross-validation, and the bias-variance tradeoff from a statistical perspective, but does not address modern deep learning regularization techniques or recent theoretical developments.

This Survey's Contributions: Our work differs from prior surveys in several key aspects:

1. We provide a unified taxonomy spanning statistical, neural network, and ensemble methods, revealing connections across traditionally separate subfields.
2. We incorporate recent theoretical developments including double descent (Belkin et al., 2019), grokking (Power et al., 2022), and sharpness-aware minimization (Foret et al., 2021) that post-date existing surveys.
3. We introduce objective-based techniques as an emerging family, connecting them to historical precursors in quantitative finance.
4. We provide practical decision guidelines grounded in systematic literature synthesis rather than anecdotal recommendations.

## 3 Methodology

This survey employs a narrative review approach to synthesize the extensive literature on overfitting mitigation techniques in machine learning. We adopt a purposive sampling strategy designed to ensure comprehensive coverage of technique families while emphasizing seminal contributions and recent advances.

A narrative review is the appropriate methodology here for several reasons. First, the techniques surveyed span fundamentally different subfields (classical statistics, deep learning, ensemble methods, information theory, quantitative finance) with incompatible evaluation protocols, datasets, and performance metrics. This heterogeneity makes formal meta-analytic pooling of effect sizes infeasible. Second, our primary goal is to unify disparate technique families under a common taxonomy and provide practical selection guidance, which requires the interpretive synthesis and cross-domain integration that narrative reviews are designed for. Third, many of the most important developments we cover (double descent, grokking, sharpness-aware minimization) are too recent and methodologically diverse for the structured inclusion/exclusion criteria of a formal systematic review to apply meaningfully. Where we describe our search strategy and inclusion approach below, we do so for transparency and reproducibility, not to claim compliance with systematic review protocols such as PRISMA.

### 3.1 Scope and objectives

This survey addresses the following questions:

1. What are the primary families of overfitting mitigation techniques, and how do they relate theoretically and practically?
2. What are the core mechanisms, strengths, weaknesses, and appropriate application domains for each technique?
3. How has the toolkit for mitigating overfitting evolved from classical statistical learning to modern deep learning?
4. What empirical evidence supports the effectiveness of different techniques across problem domains?
5. What emerging paradigms and open research questions characterize the current frontier?

### 3.2 Literature selection

We identified relevant literature through searches of major databases including IEEE Xplore, ACM Digital Library, arXiv (cs.LG and stat.ML categories), Google Scholar, and Semantic Scholar. Search terms included combinations of “overfitting,” “generalization,” “regularization,” and specific technique names (e.g., “dropout,” “batch normalization,” “weight decay,” “data augmentation”). We also conducted targeted searches for recent developments including “double descent,” “sharpness-aware minimization,” and “grokking.”

The literature coverage spans from foundational work (Tikhonov regularization; Tikhonov, 1943) through January 2026, with particular emphasis on developments since 2015 that have reshaped understanding of generalization in deep learning.

### 3.3 Inclusion approach

Papers were selected based on their contribution to understanding overfitting mitigation, prioritizing: (1) seminal papers introducing influential techniques, (2) theoretical works establishing foundational concepts, (3) empirical studies providing comparative evidence, and (4) recent papers addressing emerging phenomena. We included peer-reviewed journal articles, major conference papers, and significant preprints that have shaped the field.

The final survey synthesizes approximately 95 papers directly addressing overfitting mitigation techniques, supplemented by additional references providing theoretical background, methodological context, and historical perspective.

### 3.4 Synthesis approach

We employ a narrative synthesis organized around our five-family taxonomy. For each technique, we provide: historical context and motivation, mathematical formulation, mechanism of action, strengths and weaknesses, empirical evidence summary, appropriate application domains, and connections to other techniques. Quantitative meta-analysis was not performed due to heterogeneity in evaluation protocols, datasets, and metrics across studies.

### 3.5 Limitations

We acknowledge several limitations of this narrative approach. Publication bias toward positive results may overstate technique effectiveness. The rapid evolution of the field means some recent advances may be underrepresented. Our emphasis on English-language publications excludes potentially relevant work. The selected papers represent a purposive sample designed for comprehensive coverage rather than exhaustive enumeration; additional relevant work exists beyond those cited.

## 4 Theoretical foundations

Overfitting arises when model capacity, training dynamics, and evaluation protocols allow a learner to fit idiosyncrasies of the training set that do not persist in new data. This section summarizes the main theoretical lenses used throughout the survey. Bias-variance reasoning provides an intuitive picture of the capacity trade-off, statistical learning theory relates capacity to sample size via generalization bounds, and modern deep learning perspectives explain why overparameterized models can still generalize. We also highlight the Bayesian view that connects common regularizers to explicit prior assumptions.

### 4.1 Notation

Table 1 defines the mathematical notation used throughout this survey.

**Table 1:** Mathematical notation.

**Symbol**	**Description**
**w**	Model weight vector
L	Loss function
$L_{train},L_{val}$	Training and validation loss
λ	Regularization strength
η	Learning rate
*n*	Number of training samples
*p*	Dropout retention probability
*R*[*f*], *R*_*emp*_[*f*]	True and empirical risk
H	Hypothesis class
*d*_*VC*_	VC dimension
σ^2^	Variance/noise variance
ρ	SAM perturbation radius

### 4.2 The bias-variance decomposition

The foundational framework for understanding overfitting is the bias-variance decomposition (Geman et al., 1992). For a supervised learning problem with true function *f*(*x*) and learned estimator $\hat{f}\left(x\right)$, the expected prediction error at point *x* decomposes as:


$$\begin{array}{l} 𝔼\left[{\left(y-\hat{f}\left(x\right)\right)}^{2}\right]=\text{Bias}{\left[\hat{f}\left(x\right)\right]}^{2}+\text{Var}\left[\hat{f}\left(x\right)\right]+\sigma^{2} \end{array}$$

where:

- $\text{Bias}\left[\hat{f}\left(x\right)\right]=𝔼\left[\hat{f}\left(x\right)\right]-f\left(x\right)$ measures systematic deviation from the true function.
- $\text{Var}\left[\hat{f}\left(x\right)\right]=𝔼\left[{\left(\hat{f}\left(x\right)-𝔼\left[\hat{f}\left(x\right)\right]\right)}^{2}\right]$ measures sensitivity to training data fluctuations.
- σ^2^ is the irreducible noise variance.

Bias captures systematic error from an overly restrictive hypothesis class, while variance captures sensitivity to the particular training sample. A canonical example is polynomial regression: low-degree polynomials underfit (high bias), whereas very high-degree polynomials can fit noise and fluctuate substantially across training sets (high variance). Many interventions in this survey can be interpreted through this lens. Parameter penalties and early stopping reduce variance by constraining effective complexity, data augmentation increases the effective sample size, and ensembling reduces variance by averaging over multiple fitted models. The irreducible noise term σ^2^ remains even for an optimal model and is primarily addressed through data quality and measurement improvements.

### 4.3 Statistical learning theory

#### 4.3.1 VC dimension and generalization bounds

Vapnik-Chervonenkis (VC) theory (Vapnik, 1998) provides distribution-free generalization bounds by quantifying the capacity of a hypothesis class H through its VC dimension *d*_*VC*_, the size of the largest set of points that can be shattered by H. As a concrete example, linear classifiers in ℝ^*d*^ have *d*_*VC*_ = *d* + 1.

For a hypothesis class H with VC dimension *d*_*VC*_, with probability at least 1 − δ:


$$\begin{array}{l} R\left[f\right]\leq R_{emp}\left[f\right]+\sqrt{\frac{d_{VC}\left(\log\left(2n/d_{VC}\right)+1\right)+\log\left(4/\delta \right)}{n}} \end{array}$$

where *R*[*f*] is the true risk, *R*_*emp*_[*f*] is the empirical risk, and *n* is the sample size.

The practical takeaway is that the gap between training performance and test performance is controlled by a capacity term that scales roughly as $\sqrt{d_{VC}/n}$. When *d*_*VC*_ is large relative to *n*, even near-zero training error can be consistent with poor generalization. This motivates either increasing effective sample size (more data, augmentation, pretraining) or reducing effective capacity (regularization, pruning, depth constraints, feature selection). Because the bound is distribution-free, it can be conservative, but it captures a central lesson that recurs across technique families: generalization improves when effective capacity grows more slowly than data.

#### 4.3.2 Rademacher complexity

Where VC bounds apply uniformly across all possible datasets, Rademacher complexity adapts to the data at hand, often yielding tighter and more useful estimates. Rademacher complexity provides tighter, data-dependent bounds (Bartlett and Mendelson, 2002) by measuring how strongly a hypothesis class can correlate with random noise on the observed sample. Intuitively, if H can fit random labels easily, it can also memorize idiosyncrasies of a finite training set, increasing overfitting risk. Formally, the empirical Rademacher complexity is


$$\begin{array}{l} R_{n}\left(H\right)={𝔼}_{\sigma }\left[\underset{f\in H}{\sup}\frac{1}{n}\overset{n}{\underset{i=1}{\sum }}\sigma_{i}f\left(x_{i}\right)\right] \end{array}$$

where σ_*i*_ ∈ {−1, +1} are independent Rademacher variables.

A standard bound takes the form:


$$\begin{array}{l} R\left[f\right]\leq R_{emp}\left[f\right]+2R_{n}\left(H\right)+3\sqrt{\frac{\log\left(2/\delta \right)}{2n}} \end{array}$$

The practical implication is similar to VC bounds but often more informative in practice: regularizers that constrain norms, margins, Lipschitz constants, or hypothesis smoothness reduce $R_{n}\left(H\right)$ and therefore tighten the expected generalization gap. This lens helps explain why weight decay, margin-based losses, and certain data augmentation strategies can improve generalization even when the nominal parameter count is large.

#### 4.3.3 Stability and generalization

Generalization can also be analyzed through algorithmic stability, which measures how much a learning algorithm's output changes when a single training example is removed or replaced (Bousquet and Elisseeff, 2002). Intuitively, a stable algorithm cannot rely too heavily on any one example and is therefore less likely to memorize noise. Many interventions in this survey improve stability. Explicit regularization (e.g., weight decay) makes optimization less sensitive to individual samples, early stopping limits the degree of adaptation to the training set, and ensembling reduces sensitivity by averaging over multiple fitted models. Stability-based arguments are particularly useful when distribution-free capacity bounds are too conservative for modern deep networks.

### 4.4 PAC learning framework

The preceding bounds describe how well a given model generalizes; the PAC framework asks a more basic question: how much data does a learning algorithm need to guarantee good generalization in the first place?

The Probably Approximately Correct (PAC) framework (Valiant, 1984) formalizes learnability in terms of accuracy and confidence. For error tolerance ϵ and failure probability δ, a concept class C is PAC-learnable if there exists an algorithm A such that, for all data distributions D,


$$\begin{array}{l} P\left[R\left[f_{A}\right]-R\left[f^{*}\right]\leq \epsilon \right]\geq 1-\delta \end{array}$$

given polynomial sample complexity in 1/ϵ, 1/δ, and problem size. The value of PAC theory for overfitting is that it makes sample complexity explicit: to guarantee small generalization error with high probability, the required number of training samples must grow with hypothesis class capacity. In many classical settings, this dependence is expressed through VC dimension. For example, a standard binary classification sample complexity bound scales on the order of


$$\begin{array}{l} n=O\left(\frac{d_{VC}+\log\left(1/\delta \right)}{\epsilon^{2}}\right) \end{array}$$

up to logarithmic factors (tighter bounds with 1/ϵ dependence exist for realizable settings). While such bounds can be loose for modern deep networks, they provide a useful diagnostic: when data are scarce, controlling effective capacity (or transferring strong inductive biases via pretraining) is usually necessary to prevent memorization.

### 4.5 Modern perspectives: beyond classical theory

#### 4.5.1 The double descent phenomenon

**Figure 1:**
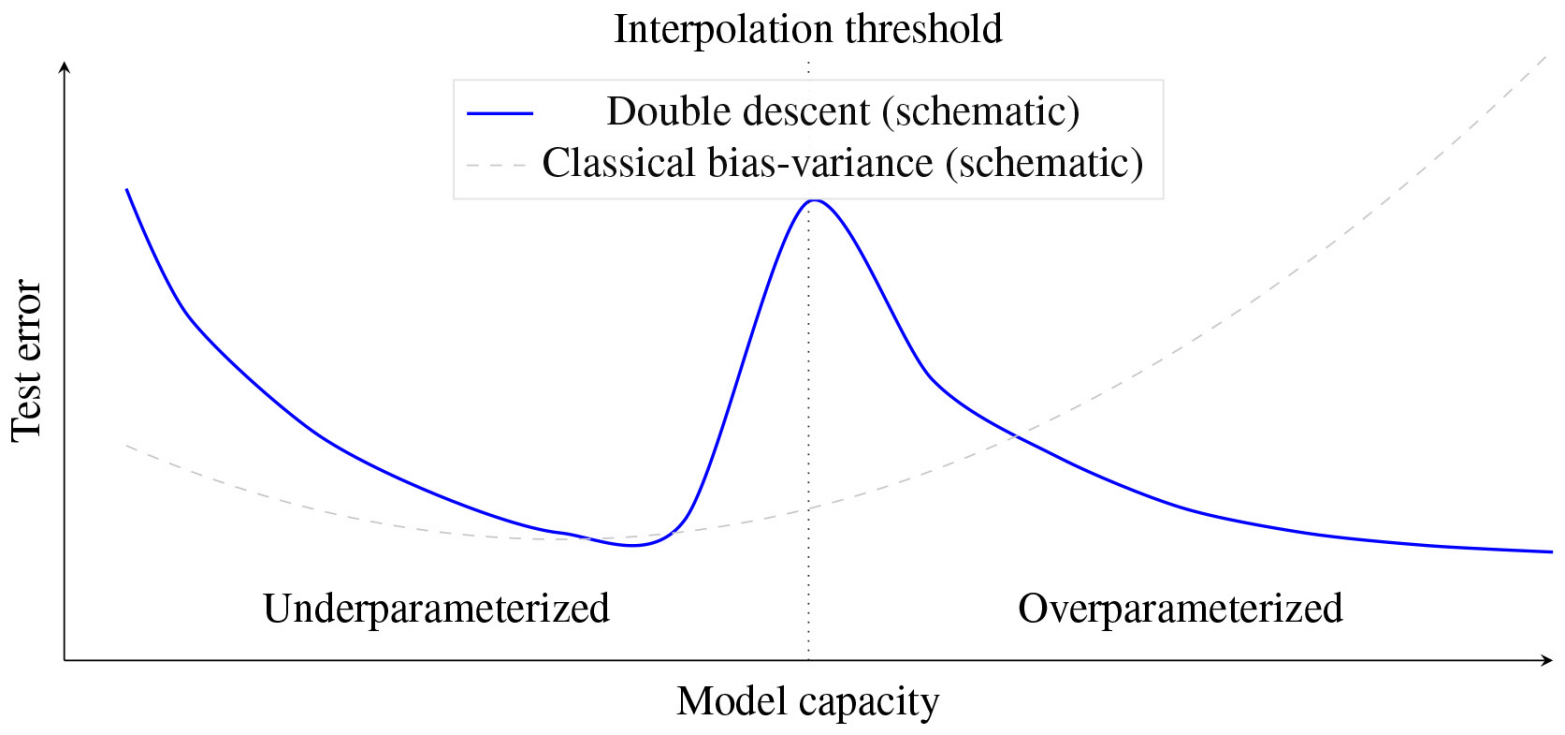
Schematic double descent: test error peaks near the interpolation threshold, then decreases again as models become highly overparameterized. The dashed curve shows the classical U-shaped bias-variance intuition.

Classical bias-variance intuition suggests that test error follows a single U-shaped curve as model capacity increases. In many modern settings, test error instead exhibits a double descent curve: it decreases with capacity, peaks near the interpolation threshold where training error approaches zero, and then decreases again as models become highly overparameterized (Belkin et al., 2019; Nakkiran et al., 2021; Figure 1).

Double descent matters for practice because it weakens the rule of thumb that larger models necessarily overfit more. The location and height of the interpolation peak depend strongly on label noise, optimization, and inductive bias (Nakkiran et al., 2021). Practitioners should therefore treat capacity control as one lever among several: increasing data quality/quantity, using augmentation, and choosing stable training dynamics can be as important as adding explicit complexity penalties.

It is worth noting how double descent relates to the VC-dimension bounds discussed in Section 4.3.1. The two are not contradictory. VC bounds are worst-case and distribution-free: they bound what *could* happen for any data distribution and any learning algorithm that selects from a hypothesis class of a given capacity. Double descent shows what *does* happen in practice when specific optimizers and architectures impose strong implicit constraints on the learned function. The key distinction is between nominal capacity (the total number of parameters, which is what VC dimension tracks) and effective capacity (the subset of functions the learning algorithm actually explores, shaped by initialization, optimization trajectory, and architectural inductive biases). In the overparameterized regime, effective capacity can be far smaller than nominal capacity, allowing interpolation without the worst-case generalization failure that VC bounds permit. For practical model selection, this means that VC-style reasoning remains a useful diagnostic for data-scarce settings where capacity clearly exceeds sample size, but it should not be the sole guide when working with modern architectures where implicit regularization plays a large role.

#### 4.5.2 Neural tangent kernels

To understand neural tangent kernels, we first review kernel methods. A kernel *K*(*x, x*′) is a similarity function that measures how alike two inputs are. The key insight is that kernels implicitly define a feature space—a (potentially infinite-dimensional) representation where each input *x* is mapped to a feature vector ϕ(*x*), and the kernel computes the inner product *K*(*x, x*′) = 〈ϕ(*x*), ϕ(*x*′)〉. This allows algorithms to work in rich feature spaces without explicitly computing the features, a technique known as the “kernel trick.” For example, a polynomial kernel *K*(*x, x*′) = (*x*^⊤^*x*′ + 1)^*d*^ implicitly computes similarities in a space of all polynomial features up to degree *d*.

In kernel regression, predictions take the form $\hat{f}\left(x\right)=\underset{i}{\sum }\alpha_{i}K\left(x,x_{i}\right)$, where the coefficients α_*i*_ are learned from data. Regularization in this setting (e.g., adding λ∥α∥^2^ to the objective) controls the smoothness and complexity of the fitted function by penalizing large coefficients.

Jacot et al. (2018) made a remarkable discovery: in the infinite-width limit, gradient descent training of a neural network is mathematically equivalent to kernel regression with a specific kernel called the Neural Tangent Kernel (NTK):


$$\begin{array}{l} K_{NTK}\left(x,x^{\prime }\right)=\nabla_{\theta }f_{\theta_{0}}{\left(x\right)}^{⊤}\nabla_{\theta }f_{\theta_{0}}\left(x^{\prime }\right) \end{array}$$

where *f*_θ_(*x*) is the network output and θ_0_ denotes the (random) initialization. The NTK measures similarity between inputs based on how similarly the network's output changes when parameters are perturbed—inputs that cause similar gradient directions are considered similar by the kernel.

This viewpoint clarifies why heavily overparameterized networks can interpolate training data without necessarily overfitting: the induced kernel and the optimization dynamics impose an implicit bias toward particular functions (specifically, functions that are “smooth” with respect to the NTK geometry), and explicit penalties or early stopping correspond to well-understood regularization mechanisms in kernel regression. Practically, NTK analyses are most relevant when training stays close to initialization (very wide networks and small learning rates). In regimes where feature learning is strong—where the network substantially changes its internal representations during training—the NTK approximation can be inaccurate, so empirical validation and careful evaluation remain essential.

#### 4.5.3 Implicit regularization

Modern deep learning often achieves good generalization without explicit regularization. This “implicit regularization” arises from:

- Optimization algorithm choice (SGD vs. Adam; Smith et al., 2021).
- Architecture inductive biases [convolutions (LeCun et al., 1998; Krizhevsky et al., 2012; He et al., 2016), attention (Vaswani et al., 2017; Devlin et al., 2019)].
- Initialization schemes (Glorot and Bengio, 2010; He et al., 2015).
- Training dynamics and early stopping (Prechelt, 1998).
- Implicit dynamical regularization from training trajectories (Bonnaire et al., 2025).

Recent work by Bonnaire et al. (Bonnaire et al., 2025; NeurIPS 2025 Best Paper) reveals that training dynamics operate on two distinct timescales: an early generalization phase whose duration is dataset-independent, followed by a later memorization phase whose duration scales linearly with dataset size. This implicit dynamical regularization explains why overparameterized models trained with standard optimization can avoid memorization—the generalization window expands with data, providing a natural form of regularization.

#### 4.5.4 Sharpness and flat minima

A growing body of work connects generalization to loss landscape geometry. Flat minima (regions where the loss varies slowly under parameter perturbations) are associated with better generalization (Hochreiter and Schmidhuber, 1997; Keskar et al., 2017). This insight motivates sharpness-aware training objectives such as SAM (Section 5.2.4), which explicitly seeks parameters that remain low-loss within a neighborhood.

### 4.6 The regularization-prior connection

Many regularization techniques have Bayesian interpretations. Consider ridge regression with objective:


$$\begin{array}{l} L\left(\text{w}\right)=∥\text{y}-\text{X}\text{w}{∥}^{2}+\lambda ∥\text{w}{∥}^{2} \end{array}$$

This is equivalent to maximum a posteriori (MAP) estimation with a Gaussian prior $\text{w}\sim N\left(0,\sigma^{2}/\lambda \text{I}\right)$. Similarly, L1 regularization corresponds to a Laplace prior, inducing sparsity.

This connection provides a principled framework: regularization encodes prior beliefs about model structure. The strength λ balances data fit against prior beliefs, with the optimal value depending on prior validity and data quantity.

Taken together, these perspectives highlight why overfitting mitigation is rarely achieved by a single ingredient. It typically requires coordinated choices that control effective capacity, shape training dynamics, increase effective sample size, and enforce evaluation integrity. The taxonomy in Section 5 organizes methods by their primary mechanism, and Section 6 translates these mechanisms into actionable selection and combination guidance.

## 5 A taxonomy of overfitting mitigation techniques

We classify techniques for mitigating overfitting into five families based on their primary mechanism of action: (i) parameter-based penalties that directly constrain weights, (ii) training-based interventions that modify learning dynamics, (iii) data-based methods that change the effective training distribution, (iv) ensemble-based methods that average over models to reduce variance, and (v) objective-based approaches that redesign the optimization target to encode generalization desiderata. Table 2 summarizes the families and typical contexts where each tends to be most useful.

**Table 2:** Overview of overfitting mitigation technique families.

**Family**	**Mechanism**	**Key techniques**	**Primary domains**	**Compute cost**
Parameter-based	Constrain parameter magnitudes via loss penalties	L1, L2, Elastic Net, weight decay	Universal	Low
Training-based	Modify learning dynamics or architecture behavior	Dropout, early stopping, batch norm, SAM	Neural networks	Low–Medium
Data-based	Enrich training distribution	Augmentation, Mixup, adversarial training	Limited data	Medium
Ensemble-based	Aggregate multiple models	Bagging, boosting, stacking	Tabular, trees	High
Objective-based	Redefine optimization target	Information bottleneck, IRM, contrastive objectives, risk-adjusted objectives	Representation learning, robustness	Low–medium

Some techniques operate at the boundary of multiple families. SAM, for example, is classified here as training-based because its defining contribution is a perturbation step that changes optimization dynamics at each iteration. However, it can also be viewed through an objective-based lens, since it reformulates the loss as a min-max problem over a neighborhood of parameters. Similarly, label smoothing modifies the supervision signal, which could be interpreted as data-based (altering the target distribution) or objective-based (changing what the loss function optimizes toward). We classify each technique by its primary mechanism, the aspect that most directly explains how and why it reduces overfitting, while noting cross-family connections in the individual technique discussions. These overlaps are not a weakness of the taxonomy; they reflect the deeper fact that many effective regularization strategies share common structure through the constrained optimization and Bayesian prior frameworks described in Section 4.

### 5.1 Parameter-based techniques

Parameter-based regularization constrains model complexity by penalizing parameter magnitudes. These techniques are among the oldest and most theoretically understood.

#### 5.1.1 L2 regularization (ridge regression/weight decay)

Historical Context: Ridge regression was introduced by Hoerl and Kennard (1970) to address ill-conditioned least squares problems. In neural networks, the equivalent technique is called weight decay (Krogh and Hertz, 1991).

Mathematical Formulation:


$$\begin{array}{l} L_{ridge}\left(\text{w}\right)=L_{data}\left(\text{w}\right)+\frac{\lambda }{2}∥\text{w}{∥}_{2}^{2}=L_{data}\left(\text{w}\right)+\frac{\lambda }{2}\underset{j}{\sum }w_{j}^{2} \end{array}$$

Mechanism: The L2 penalty shrinks all weights toward zero proportionally. The gradient update becomes:


$$\begin{array}{l} w_{j}\leftarrow w_{j}-\eta \left(\frac{\partial L_{data}}{\partial w_{j}}+\lambda w_{j}\right)=\left(1-\eta \lambda \right)w_{j}-\eta \frac{\partial L_{data}}{\partial w_{j}} \end{array}$$

The multiplicative factor (1 − ηλ) “decays” weights each step.

Theoretical Properties:

- Closed-form solution for linear models: $\hat{\text{w}}={\left({\text{X}}^{T}\text{X}+\lambda \text{I}\right)}^{-\;1}{\text{X}}^{T}\text{y}$.
- Bayesian interpretation: Gaussian prior *p*(**w**) ∝ exp(−λ∥**w** ∥^2^/2).
- Convex optimization: unique global minimum.
- Handles multicollinearity by stabilizing matrix inversion.

Strengths:

- Simple implementation; single hyperparameter.
- Well-understood theoretically.
- Effective for preventing extreme weights.
- Convex; no local minima concerns.

Weaknesses:

- Shrinks all parameters; no feature selection.
- May underfit if λ too large.
- Less effective than dropout in deep networks for some architectures.

Empirical Evidence: Zhang et al. (2017) showed weight decay provides modest but consistent improvements across CNN architectures, though its effect is less pronounced than dropout or data augmentation.

#### 5.1.2 L1 regularization (Lasso)

Historical Context: Tibshirani introduced Lasso in 1996 (Tibshirani, 1996), motivated by the desire for sparse, interpretable models.

Mathematical Formulation:


$$\begin{array}{l} L_{lasso}\left(\text{w}\right)=L_{data}\left(\text{w}\right)+\lambda ∥\text{w}{∥}_{1}=L_{data}\left(\text{w}\right)+\lambda \underset{j}{\sum }|w_{j}| \end{array}$$

Mechanism: The L1 penalty's non-differentiability at zero produces exact sparsity, meaning many weights become exactly zero. Geometrically, the constraint region's corners favor axis-aligned solutions.

Theoretical Properties:

- Bayesian interpretation: Laplace prior *p*(*w*_*j*_) ∝ exp(− λ|*w*_*j*_|).
- Convex but non-smooth; requires specialized solvers (coordinate descent, proximal methods).
- Performs automatic feature selection.

Strengths:

- Produces sparse, interpretable models.
- Automatic feature selection.
- Effective in high-dimensional settings (*p*>>*n*).

Weaknesses:

- Unstable with correlated features (arbitrarily selects one).
- Non-differentiable; requires specialized optimization.
- May over-sparsify.

#### 5.1.3 Elastic Net

Zou and Hastie (2005) combined L1 and L2 penalties:


$$\begin{array}{l} L_{elastic}\left(\text{w}\right)=L_{data}\left(\text{w}\right)+\lambda_{1}∥\text{w}{∥}_{1}+\lambda_{2}∥\text{w}{∥}_{2}^{2} \end{array}$$

This achieves sparsity while grouping correlated features, addressing Lasso's instability.

#### 5.1.4 Spectral normalization

For neural networks, Miyato et al. (2018) proposed constraining the spectral norm (largest singular value) of weight matrices:


$$\begin{array}{l} \bar{W}=W/\sigma \left(W\right) \end{array}$$

where σ(*W*) denotes the largest singular value of the weight matrix *W*.

To understand why this helps, recall that a matrix *W* transforms input vectors, and its spectral norm σ(*W*) measures the maximum factor by which *W* can stretch any input vector. When σ(*W*) is large, small changes in the input can produce large changes in the output, making the network sensitive to perturbations and potentially unstable during training.

By normalizing each weight matrix to have spectral norm equal to 1, spectral normalization ensures that each layer is 1-Lipschitz: the output cannot change faster than the input changes. This bounds the Lipschitz constant of the entire network (the product of per-layer constants), controlling how sensitive the network's predictions are to input perturbations. Networks with bounded Lipschitz constants are more robust to adversarial examples, exhibit smoother decision boundaries, and are less prone to memorizing noisy training examples. Spectral normalization is particularly important in generative adversarial networks (GANs), where training instability is a major challenge, but it also provides regularization benefits in discriminative models.

#### 5.1.5 Comparative analysis

Table 3 summarizes parameter-based techniques across three key properties:

**Table 3:** Comparison of parameter-based techniques.

**Technique**	**Sparsity**	**Stability**	**Interpretability**
L2/weight decay	No	High	Low
L1/Lasso	Yes	Low	High
Elastic Net	Partial	Medium	Medium
Spectral norm	No	High	Low

Sparsity refers to whether the technique produces models with exactly zero weights. Sparse models are often more interpretable and computationally efficient, as zero weights indicate features that can be ignored. L1 regularization produces exact sparsity due to its non-differentiable penalty at zero, while L2 regularization shrinks weights toward zero but never exactly to zero.

Stability refers to how consistently the technique selects the same features or produces similar weight patterns across different training runs or slight perturbations to the data. High stability is desirable for reproducibility and reliability. L1 regularization has low stability because, when features are correlated, it may arbitrarily select one over another depending on small data variations. L2 regularization distributes weight across correlated features, yielding more stable solutions.

Interpretability refers to how easily the resulting model can be understood by humans. Sparse models (from L1) are more interpretable because they explicitly identify which features matter. Dense models (from L2) use all features with varying weights, making it harder to identify the most important factors.

### 5.2 Training-based techniques

These techniques modify the training process itself rather than adding explicit penalties.

#### 5.2.1 Dropout

Historical Context: Srivastava et al. (2014) introduced dropout as a technique to prevent neuron co-adaptation.

Mathematical Formulation: During training, each neuron is retained with probability *p* (typically 0.5 for hidden layers):


$$\begin{array}{l} {\tilde{h}}_{i}=r_{i}\cdot h_{i},\;r_{i}\sim \text{Bernoulli}\left(p\right) \end{array}$$

where *h*_*i*_ is the original activation of neuron *i*, ${\tilde{h}}_{i}$ is the dropped-out activation, and *r*_*i*_ is a random mask variable. The Bernoulli distribution is used because it is the natural probability distribution for binary outcomes: each *r*_*i*_ independently takes value 1 (neuron retained) with probability *p*, or value 0 (neuron dropped) with probability 1 − *p*. This binary masking creates a stochastic “thinning” of the network at each training step.

At test time, activations are scaled by *p* (or equivalently, training activations scaled by 1/*p*). This scaling ensures that the expected activation magnitude at test time matches the expected magnitude during training, since 𝔼[*r*_*i*_ · *h*_*i*_] = *p* · *h*_*i*_.

Mechanism: Dropout can be interpreted as:

1. Training an ensemble of 2^*n*^ “thinned” networks (for *n* droppable units).
2. Approximate Bayesian inference over network weights (Gal and Ghahramani, 2016).
3. Noise injection forcing robust feature learning.

Variants:

- DropConnect (Wan et al., 2013): Drops connections instead of neurons.
- Spatial Dropout (Tompson et al., 2015): Drops entire feature maps in CNNs.
- DropBlock (Ghiasi et al., 2018): Drops contiguous regions.
- Variational Dropout (Kingma et al., 2015): Learns dropout rates.

Strengths:

- Highly effective regularizer for fully-connected layers.
- Implicit ensemble interpretation.
- Uncertainty quantification capability.

Weaknesses:

- Increases training time (need more epochs).
- Less effective for CNNs than spatial variants.
- Requires scaling at test time.
- Hyperparameter (dropout rate) sensitivity.

Empirical Evidence: Dropout consistently improves generalization in fully-connected networks. However, its benefits are reduced when combined with batch normalization (Li et al., 2019).

#### 5.2.2 Early stopping

Historical Context: Early stopping has long been used as a practical regularizer in neural networks (Morgan and Bourlard, 1990; Prechelt, 1998).

Mathematical Formulation: Training halts when validation loss $L_{val}^{\left(t\right)}$ fails to improve for *k* consecutive epochs:


$$\begin{array}{l} t^{*}=\arg\underset{t}{\min}L_{val}^{\left(t\right)}\;s.t.\;L_{val}^{\left(t+i\right)}\geq L_{val}^{\left(t\right)}\forall i\in \left[1,k\right]\; \end{array}$$

Mechanism: Early stopping exploits the fact that models typically fit signal before noise during training. Stopping at the validation minimum approximates optimal complexity.

Theoretical Properties: Bishop (1995) showed early stopping is equivalent to L2 regularization for linear models, with training time inversely related to regularization strength.

Strengths:

- Simple and computationally cheap.
- No additional hyperparameters beyond patience *k*.
- Reduces training time.

Weaknesses:

- Requires held-out validation set.
- Sensitive to validation set noise.
- May miss “grokking” phenomenon (Power et al., 2022).

The Grokking Challenge: Power et al. (2022) discovered that models can suddenly achieve perfect generalization long after apparent convergence, a phenomenon they termed “grokking.” This challenges early stopping's assumptions and suggests some problems require extended training beyond the validation minimum. Recent work further examines grokking dynamics and transferability in Transformers (He et al., 2026). A particularly intriguing theoretical perspective interprets grokking as a “computational glass relaxation,” where memorization resembles rapid cooling into a non-equilibrium glassy state, and generalization emerges through slower relaxation dynamics (Zhang X. et al., 2025). De Mello Koch and Ghosh (2025) argue that learning proceeds in two phases—curve fitting followed by coarse graining—providing a unifying framework connecting grokking, double descent, and the information bottleneck principle.

#### 5.2.3 Batch normalization

Historical Context: Ioffe and Szegedy (2015) introduced batch normalization to address internal covariate shift.

Mathematical Formulation: For a mini-batch B:


$$\begin{array}{l} \begin{array}{l} \mu_{B}=\frac{1}{\left|B\right|}\underset{x\in B}{\sum }x\\ \sigma_{B}^{2}=\frac{1}{\left|B\right|}\underset{x\in B}{\sum }{\left(x-\mu_{B}\right)}^{2}\\ \hat{x}=\frac{x-\mu_{B}}{\sqrt{\sigma_{B}^{2}+\epsilon }}\\ y=\gamma \hat{x}+\beta \end{array} \end{array}$$

where γ and β are learnable parameters.

Regularization Effect: While originally motivated by covariate shift, subsequent work (Santurkar et al., 2018) showed batch normalization's primary benefit is smoothing the loss landscape, enabling larger learning rates. The noise from mini-batch statistics provides implicit regularization.

Variants:

- Layer Normalization (Ba et al., 2016): Normalizes across features, suitable for RNNs/Transformers.
- Instance Normalization (Ulyanov et al., 2016): Normalizes per sample, used in style transfer.
- Group Normalization (Wu and He, 2018): Normalizes channel groups, batch-size independent.

#### 5.2.4 Sharpness-aware minimization (SAM)

Historical Context: Foret et al. (2021) introduced SAM based on the connection between flat minima and generalization.

Mathematical Formulation: SAM solves a min-max problem:


$$\begin{array}{l} \underset{\text{w}}{\min}\underset{∥\epsilon {∥}_{2}\leq \rho }{\max}L\left(\text{w}+\epsilon \right) \end{array}$$

The adversarial perturbation is approximated as:


$$\begin{array}{l} \hat{\epsilon }=\rho \frac{\nabla_{\text{w}}L\left(\text{w}\right)}{∥\nabla_{\text{w}}L\left(\text{w}\right){∥}_{2}} \end{array}$$

leading to the update:


$$\begin{array}{l} {\text{w}}_{t+1}={\text{w}}_{t}-\eta \nabla_{\text{w}}L\left({\text{w}}_{t}+{\hat{\epsilon }}_{t}\right) \end{array}$$

Mechanism: SAM seeks parameters in flat regions where loss is uniformly low, not just at a single point. This relates to PAC-Bayesian bounds connecting sharpness to generalization.

Variants:

- ASAM (Kwon et al., 2021): Adaptive, scale-invariant sharpness.
- F-SAM (Li et al., 2024): Friendly SAM with reduced stochastic noise.
- GSAM (Zhuang et al., 2022): Surrogate gap guided SAM.
- Unified SAM (Oikonomou and Loizou, 2025): Unified analysis of SAM and USAM under a single flexible update rule with convergence guarantees for non-convex problems.
- MNSAM (Kang et al., 2025): Introduces momentum acceleration to help skip sharp areas while Nesterov accelerated gradients speed convergence.

Empirical Evidence: SAM consistently improves generalization across architectures, with particularly strong results on Transformers. It doubles computation per step but often converges faster.

#### 5.2.5 Optimizer choice as implicit regularization

The choice of optimizer affects generalization through the structure and magnitude of gradient noise injected during training. SGD with mini-batches provides implicit regularization whose strength scales roughly as η/*B* (learning rate divided by batch size), helping the optimizer escape sharp minima and settle in flatter regions of the loss landscape (Smith et al., 2021). Reducing batch size or increasing learning rate amplifies this noise, which can substitute for explicit regularization in some settings.

Adaptive optimizers such as Adam (Kingma and Ba, 2015) and AdamW (Loshchilov and Hutter, 2019) change this picture in important ways. Adam maintains per-parameter learning rate estimates based on gradient moments, which rescales the effective noise differently across parameters. A practical consequence is that L2 regularization and weight decay, which are equivalent under SGD, behave differently under Adam. Loshchilov and Hutter (2019) showed that decoupled weight decay (AdamW) produces better generalization than the standard L2 penalty applied within Adam's update rule, because L2 regularization interacts with the adaptive scaling in ways that weaken its intended effect on large-gradient parameters.

Batch size also interacts with optimizer choice. Large-batch training reduces gradient noise, which can degrade generalization unless compensated by learning rate scaling (Goyal et al., 2017), explicit regularization, or techniques like SAM that directly target loss landscape geometry. The practical takeaway is that conclusions about implicit regularization are not optimizer-agnostic: a training recipe tuned with SGD may overfit when switched to Adam without adjusting regularization, and vice versa. When reporting or comparing regularization strategies, the optimizer and batch size regime should be specified.

#### 5.2.6 Label smoothing

Historical Context: Szegedy et al. (2016) introduced label smoothing as a regularization technique for training deep networks.

Mathematical Formulation: Instead of one-hot labels *y*_*k*_ ∈ {0, 1}, use smoothed labels:


$$\begin{array}{l} y_{k}^{smooth}=\left(1-\epsilon \right)y_{k}+\frac{\epsilon }{K} \end{array}$$

where *K* is the number of classes and ϵ is the smoothing parameter (typically 0.1).

Mechanism: Label smoothing prevents the model from becoming overconfident by discouraging the network from assigning full probability to any single class. This softens the decision boundary and improves calibration.

Strengths:

- Improves model calibration.
- Reduces overconfidence.
- Simple to implement; single hyperparameter.

Weaknesses:

- May hurt knowledge distillation (Müller et al., 2019).
- Optimal ϵ is problem-dependent.
- Can degrade selective classification by disrupting uncertainty rank ordering (Xia et al., 2025).

Recent Advances: Label Smoothing++ (Chhabra et al., 2025) extends the approach by learning non-target class probabilities that account for inter-class relationships. Selective Output Smoothing Regularization (SOSR; Cheng et al., 2025) generates equal logits only for overconfident samples, achieving better accuracy-calibration trade-offs than uniform smoothing.

#### 5.2.7 Cross-validation

Core Principle: Cross-validation provides unbiased estimates of generalization error and enables hyperparameter selection without a separate validation set.

*k*-Fold Cross-Validation: Partition data into *k* folds; train on *k* − 1 folds, validate on the remaining fold; repeat *k* times and average results.

Leave-One-Out (LOO): Special case with *k* = *n*; provides nearly unbiased estimates but is computationally expensive.

Nested Cross-Validation: For hyperparameter selection, use an outer loop for evaluation and inner loop for model selection, avoiding optimistic bias (Varma and Simon, 2006).

Strengths:

- Utilizes all data for both training and validation.
- Provides confidence intervals on performance.
- Essential for small datasets.

Weaknesses:

- Computationally expensive (*k*× training cost).
- May have high variance for small *k*.

#### 5.2.8 Weight averaging

Stochastic Weight Averaging (SWA; Izmailov et al., 2018): Averages weights from multiple points along the training trajectory:


$$\begin{array}{l} {\text{w}}_{SWA}=\frac{1}{T}\overset{T}{\underset{t=1}{\sum }}{\text{w}}_{t} \end{array}$$

SWA finds wider minima than standard training, improving generalization at minimal computational cost.

Exponential Moving Average (EMA): Maintains a running average of weights:


$$\begin{array}{l} {\text{w}}_{EMA}^{\left(t\right)}=\alpha {\text{w}}_{EMA}^{\left(t-1\right)}+\left(1-\alpha \right){\text{w}}^{\left(t\right)} \end{array}$$

EMA is widely used in practice (e.g., Transformers) and provides smoother, more robust models.

### 5.3 Data-based techniques

These techniques address overfitting by enriching the training distribution.

#### 5.3.1 Data augmentation

Core Principle: Apply label-preserving transformations to expand the effective training set.

Domain-Specific Augmentations:

Computer Vision:

- Geometric: rotation, scaling, flipping, cropping.
- Photometric: brightness, contrast, saturation, hue.
- Advanced: RandAugment (Cubuk et al., 2020), AutoAugment (Cubuk et al., 2019).

Natural Language Processing:

- Synonym replacement, random insertion/deletion.
- Back-translation (Sennrich et al., 2016).
- Paraphrase generation.

Audio:

- Time stretching, pitch shifting.
- SpecAugment (Park et al., 2019): masking time/frequency bands.
- Noise injection.

Strengths:

- Directly addresses data scarcity.
- Builds desired invariances.
- Computationally cheap.

Weaknesses:

- Highly domain-specific.
- Inappropriate augmentations can hurt performance.
- May create unrealistic examples.

#### 5.3.2 Mixup and variants

Mixup (Zhang et al., 2018): Creates virtual examples by linear interpolation:


$$\begin{array}{l} \begin{array}{c} \tilde{x}=\lambda x_{i}+\left(1-\lambda \right)x_{j}\\ \tilde{y}=\lambda y_{i}+\left(1-\lambda \right)y_{j} \end{array} \end{array}$$

where λ ~ Beta(α, α).

CutMix (Yun et al., 2019): Replaces rectangular regions:


$$\begin{array}{l} \begin{array}{l} \tilde{x}=\text{M}⊙x_{i}+\left(1-\text{M}\right)⊙x_{j}\\ \tilde{y}=\lambda y_{i}+\left(1-\lambda \right)y_{j} \end{array} \end{array}$$

where **M** is a binary mask and λ is the mask area ratio.

Cutout (DeVries and Taylor, 2017): Masks random regions with zeros, forcing reliance on non-occluded features.

Manifold Mixup (Verma et al., 2019): Applies mixup in hidden layer representations.

For a recent overview of mixup-style augmentations and their variants, see Jin et al. (2024).

#### 5.3.3 Adversarial training

Historical Context: Adversarial vulnerability was highlighted by Szegedy et al. (2014), motivating training procedures that explicitly optimize for worst-case perturbations.

Mathematical Formulation: Train on adversarially perturbed examples:


$$\begin{array}{l} \underset{\text{w}}{\min}{𝔼}_{\left(x,y\right)}\left[\underset{∥\delta ∥\leq \epsilon }{\max}L\left(f_{\text{w}}\left(x+\delta \right),y\right)\right] \end{array}$$

Fast Gradient Sign Method (FGSM; Goodfellow et al., 2015):


$$\begin{array}{l} \delta =\epsilon \cdot sign\left(\nabla_{x}L\left(f_{\text{w}}\left(x\right),y\right)\right) \end{array}$$

Projected Gradient Descent (PGD; Madry et al., 2018): Iterative FGSM with projection back to ϵ-ball.

Strengths:

- Primary defense against adversarial attacks.
- Improves robustness and often generalization.
- Theoretically principled.

Weaknesses:

- Computationally expensive (requires gradient computation per example).
- May reduce clean accuracy.
- Arms race with attack methods.

### 5.4 Ensemble-based techniques

Ensemble methods reduce variance by aggregating multiple models.

#### 5.4.1 Bagging (bootstrap aggregation)

Historical Context: Breiman (1996) introduced bagging to reduce variance by averaging predictors trained on bootstrap resamples.

Mathematical Formulation: Train *B* models on bootstrap samples; aggregate predictions:


$$\begin{array}{l} {\hat{f}}_{bag}\left(x\right)=\frac{1}{B}\overset{B}{\underset{b=1}{\sum }}{\hat{f}}^{\left(b\right)}\left(x\right) \end{array}$$

Variance Reduction: If base models have variance σ^2^ and pairwise correlation ρ:


$$\begin{array}{l} \text{Var}\left[{\hat{f}}_{bag}\right]=\rho \sigma^{2}+\frac{1-\rho }{B}\sigma^{2} \end{array}$$

Reducing correlation improves variance reduction.

#### 5.4.2 Random forests

Breiman's Random Forests (Breiman, 2001) extend bagging with:

- Random feature subsets at each split (decorrelates trees).
- No pruning (low bias, high variance trees).
- Out-of-bag error estimation.

Theoretical Properties:

- Consistency: converges to Bayes optimal as trees grow.
- Variable importance measures.
- Robust to hyperparameter choices.

#### 5.4.3 Boosting

Core Principle: Sequentially train weak learners to correct predecessors' errors.

AdaBoost (Freund and Schapire, 1997): Reweights examples based on previous errors.

Gradient Boosting (Friedman, 2001): Fits residuals in function space:


$$\begin{array}{l} F_{m}\left(x\right)=F_{m-1}\left(x\right)+\eta h_{m}\left(x\right) \end{array}$$

where *h*_*m*_ minimizes residuals *r*_*m*−1_ = *y* − *F*_*m*− 1_(*x*).

Modern Implementations:

- XGBoost (Chen and Guestrin, 2016): Second-order gradients, regularization.
- LightGBM (Ke et al., 2017): Gradient-based one-side sampling.
- CatBoost (Prokhorenkova et al., 2018): Ordered boosting for categorical features.

Regularization in Gradient Boosting:

- Shrinkage (learning rate η < 1).
- Subsampling (stochastic gradient boosting).
- Tree constraints (depth, leaf count).
- L1/L2 penalties on leaf values.
- Early stopping.

#### 5.4.4 Stacking

Wolpert's stacked generalization (Wolpert, 1992) trains a meta-learner on base model predictions:


$$\begin{array}{l} {\hat{f}}_{stack}\left(x\right)=g\left({\hat{f}}_{1}\left(x\right),{\hat{f}}_{2}\left(x\right),\ldots ,{\hat{f}}_{K}\left(x\right)\right) \end{array}$$

Key Considerations:

- Use cross-validation predictions to train meta-learner (avoid overfitting).
- Diverse base models improve ensemble.
- Simple meta-learners often suffice.

### 5.5 Objective-based techniques

This emerging family redefines the optimization target to directly encode generalization desiderata.

#### 5.5.1 Historical precursors: risk-adjusted ratios

Quantitative finance developed early objective-based approaches to avoid overfitting to high-variance strategies.

Sharpe Ratio (Sharpe, 1966):


$$\begin{array}{l} SR=\frac{R_{p}-R_{f}}{\sigma_{p}} \end{array}$$

Penalizes total volatility, but treats upside and downside symmetrically.

Sortino Ratio (Sortino and van der Meer, 1991):


$$\begin{array}{l} Sortino=\frac{R_{p}-R_{f}}{\sigma_{d}} \end{array}$$

Uses downside deviation σ_*d*_, recognizing upside volatility is desirable.

Calmar Ratio (Young, 1991):


$$\begin{array}{l} Calmar=\frac{R_{p}}{MaxDD} \end{array}$$

Focuses on maximum drawdown, the worst-case loss.

These ratios share a common structure: reward (numerator) divided by risk (denominator), implicitly penalizing strategies that achieve returns through undesirable risk-taking.

Related performance measures and risk estimation perspectives in portfolio theory include the Omega ratio (Shadwick and Keating, 2002) and alternative approaches to risk estimation (Biglova et al., 2004).

#### 5.5.2 The GT-score

The GT-Score (Sheppert, 2025, 2026) extends risk-adjusted thinking by incorporating statistical validity measures directly into the optimization objective:


$$\begin{array}{l} \text{GT}-\text{Score}=\frac{\mu \cdot \ln\left(z\right)\cdot r^{2}}{\sigma_{d}} \end{array}$$

where μ is mean performance, σ_*d*_ is downside deviation, *r*^2^ measures consistency over time, and ln(*z*) gates on statistical significance relative to a baseline. The intended mechanism is to penalize high-variance and unstable solutions while down-weighting improvements that are not statistically distinguishable from baseline. Reported experiments evaluate this objective across multiple optimization paradigms (e.g., random search, Bayesian optimization, genetic algorithms; Sheppert, 2026); additional independent replication would further clarify its robustness across tasks and datasets.

#### 5.5.3 Information-theoretic objectives

Information bottleneck methods (Tishby and Zaslavsky, 2015) optimize:


$$\begin{array}{l} \min I\left(X;Z\right)-\beta I\left(Z;Y\right) \end{array}$$

seeking representations *Z* that preserve task-relevant information (*I*(*Z*; *Y*)) while discarding irrelevant details (*I*(*X*; *Z*)). The parameter β controls the trade-off between compression and prediction.

Theoretical Foundation: Tishby and Zaslavsky (2015) argued that deep learning implicitly performs information bottleneck optimization, with layers progressively compressing input information while preserving label-relevant features. While this interpretation remains debated (Saxe et al., 2019), the framework provides principled guidance for representation learning.

Variational Information Bottleneck (VIB): Alemi et al. (2017) developed a variational bound enabling practical optimization:


$$\begin{array}{l} L_{VIB}=-𝔼\left[\log q\left(y|z\right)\right]+\beta \cdot KL\left(p\left(z|x\right)∥r\left(z\right)\right) \end{array}$$

where *q*, *p*, and *r* are learned distributions. VIB has shown improvements in robustness to adversarial examples and out-of-distribution generalization.

Strengths: Principled information-theoretic foundation; improves robustness; applicable across architectures.

Weaknesses: Mutual information estimation is challenging; β selection is problem-dependent; computational overhead.

#### 5.5.4 Multi-objective optimization

Generalization can be framed as multi-objective optimization (Sener and Koltun, 2018):


$$\begin{array}{l} \underset{\text{w}}{\min}\left[L_{train}\left(\text{w}\right),C\left(\text{w}\right)\right] \end{array}$$

where C measures complexity, validation loss, or other generalization proxies.

Pareto-Optimal Regularization: Rather than combining objectives with fixed weights, multi-objective approaches seek Pareto-optimal solutions where no objective can be improved without degrading another. This provides a principled alternative to manual regularization tuning.

Multi-Gradient Descent Algorithm (MGDA): Sener and Koltun (2018) proposed finding descent directions that improve all objectives simultaneously, avoiding the need to pre-specify trade-off weights.

Applications: Multi-task learning (balancing task-specific losses), neural architecture search (accuracy vs. complexity), and fairness-aware learning (performance vs. demographic parity).

#### 5.5.5 Domain-specific objective reformulations

Beyond finance, several domains have developed specialized objectives encoding generalization priors:

Healthcare—Calibration-Aware Objectives: Medical AI requires well-calibrated confidence estimates. Expected Calibration Error (ECE; Guo et al., 2017) and focal loss (Lin et al., 2017) penalize overconfident predictions:


$$\begin{array}{l} L_{focal}=-\alpha {\left(1-p_{t}\right)}^{\gamma }\log\left(p_{t}\right) \end{array}$$

The ${\left(1-p_{t}\right)}^{\gamma }$ term down-weights well-classified examples, focusing learning on uncertain cases.

NLP—Minimum Risk Training: Neural machine translation often optimizes sequence-level metrics (BLEU) rather than token-level cross-entropy (Shen et al., 2016):


$$\begin{array}{l} L_{MRT}={𝔼}_{y\sim p_{\theta }}\left[\Delta \left(y,y^{*}\right)\right] \end{array}$$

where Δ is a task-specific cost function. This aligns training objectives with evaluation metrics.

Reinforcement Learning—Robust MDPs: Robust Markov Decision Processes optimize for worst-case performance over uncertainty sets (Iyengar, 2005):


$$\begin{array}{l} \underset{\pi }{\max}\underset{P\in P}{\min}{𝔼}_{P}\left[\underset{t}{\sum }\gamma^{t}r_{t}\right] \end{array}$$

This prevents policies from overfitting to simulator dynamics.

Computer Vision—Contrastive Objectives: SimCLR (Chen et al., 2020) and related methods optimize representations to be invariant to augmentations:


$$\begin{array}{l} L_{contrastive}=-\log\frac{\exp\left(sim\left(z_{i},z_{j}\right)/\tau \right)}{\sum_{k}\exp\left(sim\left(z_{i},z_{k}\right)/\tau \right)} \end{array}$$

The objective encodes the prior that semantic content should be preserved under transformations, yielding representations that generalize across downstream tasks.

## 6 Comparative analysis and practical guidelines

### 6.1 Empirical comparison across benchmarks

Table 4 summarizes technique effectiveness across common problem types based on our literature synthesis. The ratings reflect qualitative assessment of reported results across the cited studies, not pooled effect sizes from meta-analysis. We assigned ratings by weighing the consistency of reported improvements, the breadth of empirical evidence available, and the degree of consensus across independent evaluations. For example, dropout receives a “+++” rating for image classification based on consistent improvements reported across CNN architectures (Srivastava et al., 2014; Zhang et al., 2017), while its “+” for tabular data reflects limited evidence of benefit in that setting. Because evaluation protocols, datasets, and metrics vary substantially across studies, these ratings should be treated as informed guidance rather than precise quantitative rankings.

**Table 4:** Technique effectiveness across problem types (based on literature synthesis).

**Technique**	**Image class**.	**NLP**	**Tabular**	**Time series**	**Small data**
L2/weight decay	++	++	+++	++	++
L1/Lasso	+	+	+++	++	++
Dropout	+++	++	+	+	++
Early stopping	++	++	++	++	+++
Batch norm	+++	+	+	+	++
SAM	+++	+++	++	++	++
Data augmentation	+++	++	+	++	+++
Mixup/CutMix	+++	+	+	+	++
Adversarial training	++	++	+	+	+
Random forests	+	+	+++	++	++
Gradient boosting	+	+	+++	+++	++
Stacking	++	++	+++	++	+

### 6.2 Overfitting diagnostic checklist

Before applying regularization, practitioners should confirm that poor generalization is driven by overfitting rather than by distribution shift, label noise, or evaluation leakage. The following checklist is designed as a fast, reproducible triage that complements the decision framework below.

#### 6.2.1 Evaluation integrity (first priority)

1. Verify split correctness: ensure that the train/validation/test split reflects the intended deployment distribution and that no post-split transformations leak information across partitions.
2. Eliminate leakage and near-duplicates: deduplicate examples, enforce group splits for correlated samples (e.g., patient-level splits), and audit feature pipelines for target leakage.
3. Separate tuning from final testing: reserve an untouched test set and treat the validation set as part of model selection to avoid optimistic bias (Varma and Simon, 2006).

#### 6.2.2 Learning dynamics (second priority)

1. Inspect learning curves: a widening train–validation gap suggests classic overfitting, while both losses high indicates underfitting or optimization issues.
2. Stress-test hyperparameters: if performance is highly sensitive to small hyperparameter changes, prioritize simpler models and stronger evaluation (more folds/repeats).
3. Check calibration and stability: evaluate not only accuracy but also calibration (Guo et al., 2017) and the variance of performance across random seeds.

#### 6.2.3 Intervention selection (third priority)

1. Start with low-risk fixes: early stopping, weight decay, and basic augmentation often deliver large gains at minimal complexity.
2. Add complementary techniques: combine one technique from different families (e.g., weight decay + augmentation + ensembling) rather than stacking many within the same family.
3. Measure with robust baselines: compare to simpler baselines and report confidence intervals when feasible.

### 6.3 Decision framework

We propose the following decision framework for technique selection:

#### 6.3.1 By data availability

Very Limited Data (*n* < 1, 000):

1. Data augmentation (highest priority).
2. Strong regularization (L2, dropout with high rate).
3. Transfer learning/pre-trained models.
4. Early stopping with small patience.

Moderate Data (1, 000 < *n* < 100, 000):

1. Moderate regularization.
2. Dropout (0.2–0.5).
3. Data augmentation.
4. Cross-validation for hyperparameter selection.

Large Data (*n* > 100, 000):

1. Light regularization may suffice.
2. Focus on architecture and optimization.
3. SAM for additional gains.
4. Ensembles if computational budget allows.

#### 6.3.2 By model type

Linear Models:

- L2 for multicollinear features.
- L1/Elastic Net for feature selection.
- Cross-validation for λ selection.

Tree-based Models:

- Gradient boosting with early stopping.
- Tree depth/leaf constraints.
- Subsampling (row and column).

Neural Networks:

- Weight decay + Dropout (standard combination).
- Batch/Layer normalization.
- Data augmentation.
- SAM for improved results.

Transformers/LLMs:

- Layer normalization.
- Dropout (attention and feed-forward).
- Weight decay.
- Learning rate warmup and decay.

Foundation Models (Fine-tuning):

- Parameter-efficient fine-tuning (LoRA; Hu et al., 2022; DoRA; Liu et al., 2024) as structural regularization that limits the degrees of freedom updated during adaptation.
- Low learning rates with warmup, since the pre-trained weights already encode strong priors and aggressive updates can destroy useful representations.
- Weight decay (typically light, e.g., 0.01–0.1), applied to fine-tuning parameters.
- Early stopping on the fine-tuning validation set, with short patience given the risk of rapid overfitting on small task-specific datasets.
- Data augmentation, which becomes especially important when fine-tuning data is scarce relative to model capacity.

The pre-trained weights themselves act as a strong regularizing prior, analogous to a Bayesian prior informed by large-scale data. This changes the regularization calculus: the primary risk is not that the model lacks capacity, but that fine-tuning overwrites general-purpose representations with task-specific noise. Practitioners should monitor for catastrophic forgetting (a form of overfitting to the new task at the expense of pre-trained knowledge) and consider whether full fine-tuning, PEFT, or a hybrid approach best fits the available data and compute budget.

#### 6.3.3 By computational budget

Low Budget:

- Early stopping (saves compute).
- Weight decay.
- Basic data augmentation.

Medium Budget:

- Dropout.
- Hyperparameter tuning via cross-validation.
- Advanced augmentation.

High Budget:

- SAM (2 × compute per step).
- Ensembles.
- Neural architecture search with regularization.
- Adversarial training.

### 6.4 Common pitfalls

1. Over-regularization: Excessive regularization causes underfitting. Monitor training loss; if it plateaus high, reduce regularization.
2. Incompatible Combinations: Batch normalization + dropout can conflict (Li et al., 2019). Layer normalization is often preferred with dropout.
3. Ignoring Double Descent: In the overparameterized regime, traditional complexity penalties may hurt. Consider whether you're before or after the interpolation threshold.
4. Validation Set Contamination: Data augmentation should be applied only to training data. Augmenting validation/test sets inflates performance estimates.
5. Early Stopping Too Early: Grokking suggests some problems benefit from extended training. Use sufficient patience.
6. Neglecting Data Quality: No regularization fixes poor data. Prioritize data cleaning and curation.

### 6.5 Synergistic combinations

Certain technique combinations are particularly effective:

- Weight Decay + Dropout + Augmentation: Standard deep learning recipe.
- SAM + Strong Augmentation: State-of-the-art image classification.
- Gradient Boosting + Early Stopping + Subsampling: Robust tabular learning.
- Mixup + Label Smoothing: Improved calibration and generalization.
- Pre-training + Light Fine-tuning Regularization: Transfer learning best practice.

### 6.6 Scenario-based recommendations

To make the decision framework more concrete, Table 5 maps common real-world scenarios to recommended technique combinations. Each scenario reflects constraints that practitioners frequently encounter, and the recommendations draw on the empirical evidence and mechanism analyses presented in Sections 5 and 6.

**Table 5:** Recommended technique combinations for common real-world scenarios.

**Scenario**	**Recommended techniques**	**Primary families**	**Rationale**
Small medical dataset (*n* < 500), noisy labels	Transfer learning from pre-trained model + strong augmentation + label smoothing (ϵ = 0.1) + early stopping (patience 5–10)	Data, training, parameter	Limited data requires strong priors via pre-training; augmentation increases effective sample size; label smoothing dampens noise influence; early stopping prevents memorization
Large-scale image classification (*n* > 100 k)	SAM + CutMix/Mixup + weight decay (λ = 0.01-0.05) + cosine learning rate schedule	Training, data, parameter	Data abundance reduces overfitting risk; SAM targets flat minima for additional generalization gains; CutMix builds local invariances; light weight decay sufficient
Tabular data with many correlated features	Gradient boosting (XGBoost/LightGBM) + early stopping + column subsampling (0.5–0.8) + Elastic Net for feature pre-selection	Ensemble, training, parameter	Tree ensembles dominate tabular benchmarks; column subsampling decorrelates trees; Elastic Net handles multicollinearity for initial feature screening
Fine-tuning a foundation model on limited task data (*n* < 5 k)	LoRA/DoRA (rank 8–64) + low learning rate (1 × 10^−5^-5 × 10^−5^) with warmup + weight decay (0.01) + early stopping (patience 3–5)	Parameter, training	PEFT constrains degrees of freedom; low learning rate preserves pre-trained representations; early stopping critical given rapid overfitting risk
Time series forecasting with regime changes	Gradient boosting with temporal cross-validation + moderate weight decay + recent-window emphasis + ensemble of models across time windows	Ensemble, parameter, training	Temporal structure requires time-aware validation splits; ensembling across windows captures regime diversity; weight decay stabilizes parameter estimates
NLP classification with class imbalance	Pre-trained language model + focal loss + dropout (0.1–0.3) + back-translation augmentation + weight decay	Objective, training, data, parameter	Focal loss addresses class imbalance by down-weighting easy examples; dropout prevents co-adaptation; back-translation enriches minority class examples

## 7 Emerging trends and future directions

The overfitting landscape continues to shift as models scale, training data diversify, and deployment settings become more complex. Recent work increasingly emphasizes not just adding stronger regularizers, but understanding when modern training dynamics lead to benign interpolation, how transfer learning changes the degrees of freedom that can overfit, and how automated model selection can itself overfit evaluation protocols.

### 7.1 Current research frontiers

#### 7.1.1 Understanding overparameterization

Classical learning theory often motivates explicit capacity control: as hypothesis classes grow richer, overfitting risk should increase unless sample size grows commensurately. Modern deep networks complicate this picture. They can fit random labels (Zhang et al., 2017) yet still generalize well when trained with SGD and contemporary architectures. Work on double descent and benign overfitting reframed interpolation as a regime change rather than an inevitable failure (Belkin et al., 2019; Nakkiran et al., 2021), and recent analyses continue to map when and how double descent emerges under noise and training-time dynamics (Kubo et al., 2026). The practical implication is that overfitting is shaped as much by data quality, optimization, and inductive bias as by parameter count alone.

Key questions include:

- What implicit biases of optimization and architecture select generalizing solutions?
- How do label noise and data regimes shift the interpolation peak?
- Can we predict or detect the interpolation threshold during training?

#### 7.1.2 Foundation models and transfer learning

Pre-trained foundation models change the regularization landscape by moving many applications into a fine-tuning regime where the base model acts as a strong prior. Fine-tuning can overfit quickly when task data are limited, but overly aggressive regularization can also erase useful representations or harm calibration. Parameter-efficient fine-tuning (PEFT) methods provide a structural form of regularization by limiting the degrees of freedom updated during adaptation, as in LoRA (Hu et al., 2022) and DoRA (Liu et al., 2024).

Open challenges include:

- Fine-tuning requires different regularization than training from scratch.
- Catastrophic forgetting is a form of overfitting to new tasks.
- Choosing between full fine-tuning, PEFT, and hybrid approaches under compute and data constraints.

#### 7.1.3 Neural architecture search

Neural architecture search (NAS) introduces a second-level optimization problem: selecting architectures and hyperparameters to maximize validation performance. This creates a direct route to overfitting the validation set and to reporting optimistic results if search budgets, randomness, and evaluation protocols are not controlled (Yang et al., 2020). Mitigation parallels standard overfitting hygiene, such as nested evaluation, explicit budget reporting, and restricting search space complexity.

Key issues include:

- Validation-set overfitting when the same split drives repeated search decisions.
- Limited transferability of discovered architectures across datasets and data regimes.
- Multi-objective search (accuracy vs. complexity, stability, or calibration) as regularization.

#### 7.1.4 Continual learning

Continual learning and other non-stationary settings introduce a distinct generalization challenge: fast adaptation to recent data can resemble overfitting to the latest batches, while performance on earlier tasks degrades through forgetting. Recent surveys synthesize replay-based, regularization-based, and architectural approaches for mitigating forgetting (Sha et al., 2024).

Core challenges include:

- Preventing overfitting to recent data.
- Balancing plasticity and stability.
- Replay and regularization-based approaches.

### 7.2 Promising directions

#### 7.2.1 Automated regularization selection

Automated selection treats regularization as a first-class optimization target, aiming to learn schedules and combinations that generalize across datasets and architectures rather than relying on manual trial-and-error. Meta-learning approaches explore:

- Learning regularization schedules.
- Architecture-specific regularization.
- Data-dependent regularization strength.

#### 7.2.2 Causal regularization

When poor generalization is driven by spurious correlations that change across environments, causal regularization aims to learn predictors tied to invariant structure rather than correlational shortcuts. Directions include:

- Invariant risk minimization (Arjovsky et al., 2019).
- Causal representation learning.
- Domain generalization (Li et al., 2025).

#### 7.2.3 Uncertainty-aware objectives

Uncertainty-aware objectives seek to reduce overconfidence and improve decision-making under limited data by modeling uncertainty explicitly. Examples include:

- Bayesian deep learning.
- Conformal prediction.
- Calibration-aware training.

#### 7.2.4 Knowledge distillation as regularization

Teacher-student frameworks provide implicit regularization by constraining a student's function class toward a smoother teacher signal (Hinton et al., 2015). Common mechanisms include:

- Soft labels encode class relationships.
- Self-distillation improves generalization.
- Connection to label smoothing.
- Temporally adaptive interpolation to prevent mode collapse (Shing et al., 2025)

Recent advances include incorporating pre-training data during fine-tuning—including just 1% of pre-training data can shield models from forgetting and mitigate overfitting (Apple Machine Learning Research, 2025).

### 7.3 Open problems

1. Unified Theory: A comprehensive theory explaining when and why different regularization approaches work remains elusive.
2. Optimal Combination: Principled methods for combining techniques beyond trial-and-error are needed.
3. Generalization Metrics: Better proxies for generalization that can be computed during training would enable more targeted regularization.
4. Distribution Shift: Current techniques assume training and test distributions match. Robust generalization under distribution shift is an open challenge.
5. Efficiency: Many effective techniques (SAM, ensembles, adversarial training) are computationally expensive. Efficient approximations are needed.

## 8 Conclusion

Overfitting remains a central challenge in machine learning, but six decades of research have produced a rich arsenal of mitigation strategies. This survey has provided a comprehensive review of overfitting mitigation techniques, organized into five families: Parameter-based, Training-based, Data-based, Ensemble-based, and Objective-based.

Our key findings include:

1. No Universal Solution: Each technique family has distinct strengths and weaknesses. Effective generalization typically requires combining complementary approaches.
2. Context Dependence: Optimal technique selection depends on problem type, data availability, model architecture, and computational constraints.
3. Evolving Understanding: Recent discoveries such as double descent, grokking, and the success of overparameterization challenge classical intuitions and suggest our theoretical understanding remains incomplete.
4. Emerging Paradigms: Objective-based techniques that directly encode generalization desiderata represent a promising frontier, building on insights from risk-adjusted metrics in finance.
5. Practical Synergies: Certain technique combinations (weight decay + dropout + augmentation; SAM + strong augmentation; gradient boosting + early stopping) have emerged as particularly effective.

As machine learning systems are deployed in increasingly high-stakes domains, the importance of generalization will only grow. We hope this survey provides researchers and practitioners with the theoretical foundations, practical guidelines, and inspiration needed to build models that truly generalize.
